# Reasons behind high rate of non-compliance to scheduled office visits in hypertensive patients: results from the Egyptian registry of specialized hypertension clinics

**DOI:** 10.1186/s43044-022-00285-7

**Published:** 2022-05-31

**Authors:** Ghada Youssef, Marwa Mohamed, Magdy Abdel Hamid, Dalia El Remisy

**Affiliations:** 1 Cardiovascular Department, Kasr Al Ainy School of Medicine, Cairo University, Cairo, Egypt; 2 National Heart Institute, Giza, Egypt

**Keywords:** Hypertension, Compliance, Office visits

## Abstract

**Background:**

Hypertensive patients’ compliance to the clinic’s follow-up visits is associated with a better blood pressure control. The aim of this study was to detect the reasons of non-compliance to office visits in Egyptian hypertensive patients.

**Results:**

This is an observational, prospective, cross-sectional research study where patients were enrolled from the registry of the specialized hypertension clinics of 9 university hospitals. Those who attended less than 3 office visits, throughout the registry period, were considered non-compliant and were contacted through the phone. A simple questionnaire was prepared, which included questions about the reasons of non-compliance to follow up. There were 3014 patients eligible for inclusion in this study but only 649 patients (21.5%) completed the questionnaire. Patients claimed that the reasons of non-compliance to the follow up visits in the specialized hypertension clinics were as follows: 444 patients (68.4%) preferred to follow up elsewhere mostly in pharmacies, 53 patients (8.2%) claimed that the healthcare service was unsatisfactory, 94 patients (14.5%) were asymptomatic, and 110 patients (16.9%) said that the clinic was far from their homes. Despite non-compliance to office visits, 366 patients (59.2%) were compliant to their antihypertensive medications and 312 (48.1%) patients were compliant to salt restriction. About 34% of patients used herbs, mainly hibiscus, as adjuvant to their antihypertensive medications.

**Conclusions:**

Reasons for non-compliance to office visits in hypertensive patients were either patient-related, or healthcare-related. To improve patients’ compliance, physicians need to educate their patients about hypertension, patients need to follow their doctors’ instructions as regard medications, salt restriction and scheduled office visits, and governments need to provide better and cheaper healthcare services.

## Highlights

- Prevalence of hypertension is high, yet control rates are still low.
- The national Egyptian Specialized HTN Clinics were initiated in 2015 to improve HTN management.
- The patients’ follow up rates in the clinics were low despite uncontrolled hypertension.
- Non-attendance was due to absence of symptoms and long waiting times at clinics.
- Raising awareness about value of regular follow up is mandatory to ensure proper HTN control.

## Background

Hypertension (HTN) is a worldwide public health problem characterized by a high prevalence and high risk of vascular complications. It was shown that proper control of HTN could reduce the risk of cardiovascular mortality as well as the risk of myocardial infarction, heart failure and stroke [1]. There are many factors that contribute to the proper control of BP, including: type, number and doses of antihypertensive medications, patients’ adherence to therapy and salt restriction and patients’ compliance to follow up visits [2, 3].

HTN in Egypt is highly prevalent, poorly diagnosed, and poorly controlled [4, 5]. Many hypertensive Egyptians were not aware about having HTN and most of them presented late with complications attributed to their uncontrolled blood pressure (BP) [5].

The Egyptian Hypertension Society (EHS) launched the specialized HTN clinics (SHCs) project in 2015, in nine university hospitals of different Egyptian governorates, aiming at improving diagnosis and management of HTN as well as obtaining an updated data about HTN in Egypt [6]. In a recently published data from the Egyptian SHCs registry, it was found that after the first office visit, only 25.1% of hypertensive patients attended a second office visit, and the percentage became less in the subsequent visits [7]. As it was previously shown that compliance to scheduled clinic visits was associated with better control of HTN in hypertensive patients [8], this study aims to assess the reasons behind the low follow up rates among hypertensive Egyptians who attended the SHCs in an attempt to understand the problem and propose the proper solutions.

### Population of the study

Hypertensive patients (whether their blood pressure was controlled or not) who presented to the SHC at the assigned university hospital and whose data was recorded on the online SHC registry website in the period between January 2015 and December 2017.

### Exclusion criteria

Patients who refused to participate and those who were uncooperative or difficult to communicate with.

## Methods

### Study design

The study protocol was approved by the Ethics Committee of the Faculty of Medicine, Cairo University in March 2019. An informed consent for data collection was taken from all participants.

This is an observational, prospective, cross-sectional research studying hypertensive patients from the online registry of the SHCs of nine university hospitals in nine Egyptian governorates (Great Cairo including Cairo, Ain Shams and Helwan university hospitals, Alexandria, Tanta, Beni-Suef, Suez Canal, Menia and Assiut university hospitals).

SHCs were initiated in 2015 and the registry was active since then till December 2017. There were 2 types of collected data in this study; the first was from the online case report form (CRF) which included patients’ demographics and clinical data as well as patients’ contact information and number of clinic visits. The second type of data was derived from contacting patients through the phone and asking them to answer a questionnaire. Only patients who attended less than 3 clinics’ visits (along the 3 years of the registry) were telephoned and were asked to answer a structured phone-based questionnaire to determine the reasons of non-adherence to their follow up visits, while patients who used to come regularly at their scheduled dates (3 or more clinic visits along the 3 years of the registry) were considered compliant to follow up and were not telephoned.

### Data retrieved from the patients’ online CRF records

#### Personal data

Age (at the time of the first visit), gender, educational background including: being illiterate, reads and writes (defined as primary/preparatory school graduate or drop-out), pre university or university graduate. Employment status (employed or unemployed) and patient’s residence (urban or rural) were also reported.

#### Patients’ co morbidities and risk factors assessment

These were diagnosed either by history of drug administration for the specific disease or by laboratory workup after the first office visit. Comorbidities included: diabetes Mellitus (DM), current smoking (ex-smokers were defined as abstinence from cigarettes for more than a year), chronic kidney disease (CKD), obesity (defined as a BMI ≥ 30 kg/m^2^), coronary artery disease (CAD), heart failure (HF), stroke, and positive family history of HTN. Patients were considered to have a sedentary lifestyle when they were either elderly (> 75 years), could not move due to a neurological and/or an orthopaedic problem or had an occupation of a sedentary nature e.g. office job with long office hours and drivers.

#### Last known blood pressure reading

The average systolic and diastolic blood pressures were derived from the last recorded office visit. The aim was to identify patients who missed their scheduled next clinic visits despite having uncontrolled HTN.

#### Total number of visits to the SHC

The total number of office visits throughout the registry period were derived from the patients’ records.

### Data obtained by contacting patients through the phone

Patients who were not compliant to their office visits were telephoned and those who answered the phone were asked about the reasons why they did not attend their scheduled clinic’s visits.

We proposed reasons of inability to reach the patients, and these included: presence of invalid or wrong phone number, patient’s death, phone rang but there was no answer (in at least two different occasions), phone was closed or not available (in at least two different occasions), or others.

Those who answered the phone were asked the following questions:

- *Question 1:* Did the patient measure his/her blood pressure elsewhere? (The answer was either yes or no) and where exactly? Patients who answered this question by (yes) were asked to choose one of the options of the location where they measured their BP: pharmacy, ministry of health (MOH) hospital, insurance hospital, private doctor or at home using a home device.
- *Question 2:* If the patient was not measuring his/her BP elsewhere, then why did he/she stop coming for follow up? This question focused on three main factors that hindered clinic follow up and these were: (a) *Health care related factors* (patient wasn’t satisfied by the services offered by the clinic or patient wasn't taking medications from the clinic), (b) *Patients’ related factors*: which included; (i) patients’ awareness of the nature of HTN and its related complications (patient thought that hypertension wasn’t a serious disease or patient was asymptomatic), (ii) socio-economic and environmental factors (the clinic was far from patient’s home or financial reasons), (iii) perceived effectiveness of treatment: patient thought that antihypertensive medications would work with no need to follow up, (iv) patient followed up his/her blood pressure while following up comorbid diseases in the general medicine clinic, (v) inconvenient time of the clinic, that interfered with patients’ working hours.
- *Question 3:* Was the patient consuming any herbals aiming at reducing their uncontrolled HTN? And what kind of herbals were they using?
- *Question 4:* Was the patient compliant to his/her antihypertensive medications? Those who answered this question by (no) were asked why they weren’t compliant, and the proposed answers included the following reasons: (a) patient was asymptomatic, (b) patient experienced drug side effects, (c) patient was dependent on herbal medications for treatment of HTN.
- *Question 5:* Was the patient compliant to salt restriction?
- *Question 6:* patient was asked if he/she thought that following up their HTN was important and if they were willing to resume the clinics’ visits.

### Statistical analysis

Data was tabulated and analysed using an SPSS 26 software program. Categorical data was presented as numbers and percentages, and continuous data was presented as mean and standard deviation. Chi square test was used to compare a 2 × 2 categorical data, while comparison of means was done using a student-t test. A *p* value < 0.05 was considered significant.

## Results

**Fig. 1:**
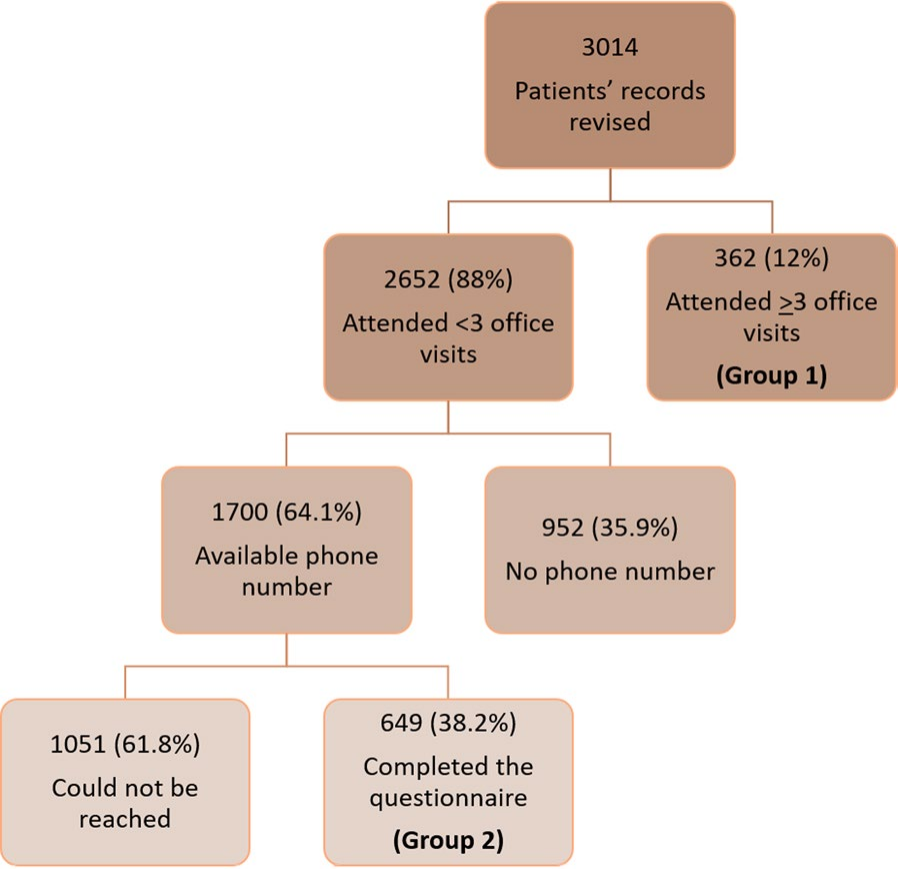
Flow chart demonstrating the inclusion of patients in the study

There were 3014 registry patients eligible to be included in the study, of whom, only 362 patients (12%) attended 3 or more clinic’s visits, Fig. 1. As shown in Fig. 1, patients who were not compliant to follow up visits (*n* = 2652, 88%) were eligible to approach through their phone contacts provided in the registry. Of those, only 1700 patients (64.1%) had available phone numbers. About 1051 patients (61.8%) could not be reached, and Table 1 demonstrates the reasons why. Invalid phone numbers were either no longer belonged to the patient (*n* = 212, 20.2%), phone number provided by the patient was no longer in service (*n* = 199, 18.9%) or despite calling several times there was no answer (*n* = 271, 25.8%) or number was closed or not available (*n* = 235, 22.4%).

**Table 1:** Reasons why patients couldn't be reached through the phone

Reasons why patients couldn't be reached	No. (%) (no. = 1051)
Invalid number	917 (87.3)
Patient died	104 (9.9)
Patient refused to answer or didn't complete the questionnaire	30 (2.9)

The last known average systolic and diastolic blood pressures of the registry patients (*n* = 3014) were 143.8 ± 21.5 mmHg and 88.4 ± 12.7 mmHg, respectively. Based on the last measured BP, 1272 patients (42.2%) had controlled BP and the remaining 1742 patients (57.8%) had uncontrolled HTN. Despite having uncontrolled HTN, 1585 patients (out of 1742, 91.0%) were not compliant to their scheduled follow up visits (*p* value  < 0.001).

Data acquired from the registry was used to compare the profile of the compliant (group 1, *n* = 362) versus the telephoned non-compliant patients (group 2, *n* = 649), Table 2. As shown in this table, compliant patients were older, with a higher female preponderance, and higher prevalence of illiteracy, unemployment, and active lifestyle. Non-compliant patients showed higher prevalence of CAD, HF, stroke and positive family history of HTN. Systolic and diastolic blood pressures were higher in the non-compliant patients’ group.

**Table 2:** Comparison between the compliant and the non-compliant patients’ profiles

Variable	Compliant pts, (Group 1) No. = 362	Non-compliant pts, (Group 2) No. = 649	*p* value
Age, years	57.1 ± 11.3	54.8 ± 11.6	0.006
Sex, male	154 (42.5)	320 (49.3)	0.039
Education, illiterate	192 (53.0)	257 (39.6)	< 0.001
Occupation, unemployed	230 (63.5)	383 (59.0)	< 0.001
Residence, urban	280 (77.3)	477 (73.5)	0.176
BMI, Kg/m^2^	33.3 ± 6.7	33.2 ± 7.0	0.864
Obesity	243 (67.1)	423 (65.2)	0.531
DM	119 (32.9)	218 (33.6)	0.817
CKD	25 (6.9)	31 (4.8)	0.156
Stroke	27 (7.5)	52 (8.0)	0.389
CAD	49 (13.5)	191 (29.4)	< 0.001
Heart Failure	26 (7.2)	80 (12.3)	0.029
Current Smokers	70 (19.3)	138 (21.3)	< 0.001
Sedentary lifestyle	63 (17.4)	332 (51.2)	< 0.001
FH of HTN	164 (45.3)	394 (60.7)	< 0.001
Last known SBP, mmHg	137.2 ± 17.9	142.4 ± 20.7	< 0.001
Last known DBP, mmHg	84.3 ± 10.9	88.2 ± 12.3	< 0.001
Uncontrolled HTN	157 (43.4)	369 (56.9)	< 0.001

Data is presented either as number (%) or mean ± standard deviation.

BMI, body mass index; CAD, coronary artery disease; CKD, chronic kidney disease; DBP, diastolic blood pressure; DM, diabetes mellitus; FH, family history; HTN, hypertension; SBP, systolic blood pressure

Patients who were successfully contacted and agreed to answer the questionnaire (*n* = 649) had many reasons for non-compliance to office visits, as demonstrated in Table 3 and some patients reported more than one reason for non-compliance.

**Table 3:** Reasons why patients stopped coming for follow up in the HTN clinic

Reasons for non-compliance to office visits	No. (%) (no. = 649)
Patient was measuring his/her BP elsewhere	444 (68.4)
- Pharmacy	239 (36.8)
- MOH hospitals	78 (12.0)
- Insurance hospitals	6 (0.9)
- Private doctor	62 (9.6)
- Home device	66 (10.2)
*Healthcare related factors	
- Healthcare service unsatisfactory	53 (8.2)
- No one called the patient to come for follow up	5 (0.7)
- Patient referred to clinic for other reasons	31 (4.8)
- Patient is not taking medications from the clinic	24 (3.7)
*Patient’s related factors	
1. Patient’s awareness	
- Patient thought that HTN is not a serious disease	0 (0)
- Patient is asymptomatic	94 (14.5)
2. Socio-economic factors	
- Clinic was far from home	110 (16.9)
- Financial reasons	24 (3.7)
- Depression	4 (0.6)
- Patient disability and inability to move easily	22 (3.4)
3. Perceived effectiveness of treatment	
- Patient is convinced that anti HTN treatment will work with no need to follow up	16 (2.5)
4. Patients follow up HTN while following up other co morbidities	35 (5.4)
5. Inconvenient time of the clinic	49 (7.6)
**Others**	
- Patient BP is controlled	71 (10.9)
- Patient was found to be normotensive	31 (4.8)
- BP elevation was situational	28 (4.3)
- Other causes	180 (27.7)

BP, blood pressure; HTN, hypertension; MOH, Ministry Of Health

Most patients (*n* = 444, 68.4%) preferred to measure their BP elsewhere and the majority of them preferred pharmacies.

Some patients (*n* = 53, 8.2%) were not satisfied by the medical service provided by the clinic because of cancellation of the clinic appointment without notice, absence of attending physicians, different attending physicians, patient thought the physician was not skilled enough, treatment prescribed by the clinic did not improve patient's symptoms, there was no comprehensive examination but only measurement of blood pressure and indifferent or uncaring physician’s attitude towards the patient. Over crowdedness and long waiting time were other factors that made patients unsatisfied with the healthcare service which accordingly affected patients’ compliance.

Some patients (*n* = 110, 16.9%) claimed that the reason for not following up was because the clinic was far from where they reside, while others did not come because they moved to another governorate or travelled abroad.

As regard financial reasons: some patients could not afford the cost of transportation to the clinic and the cost of investigations and treatment.

Some patients (*n* = 35, 5.4%) followed up their hypertension while following up other co morbidities in the general clinic and this was specifically evident in patients with CKD who followed up their blood pressure at the nephrology clinic.

The SHC’s time was inconvenient to some patients as they could not take time out of work, child or sick care or other important medical procedures (e.g. dialysis session).

Some patients (*n* = 31, 4.8%) were referred to the SHCs for control of BP prior to certain investigation or procedures (coronary angiography, perfusion scan, stress electrocardiography, and surgery).

Some patients (*n* = 22, 3.4%) could not move easily due to either an orthopaedic problem e.g. osteoarthritis, a residual weakness from a previous stroke or because of being old and fragile. Going to the clinic was considered a burden for these patients and their families, so they stopped coming. Other patients were either depressed due to loss of a family member or frustrated due to failure to achieve the blood pressure control goal.

After excluding patients who were found to be normotensive (wrong diagnosis of HTN, *n* = 31), in the next question, the remaining patients (*n* = 618) were asked about compliance to antihypertensive medications and to salt restriction. Of those, 366 patients (59.2%) were compliant to their medical treatment despite noncompliance to the follow up visits. About one fifth (*n* = 51, 20.2%) of the patients who were not compliant to their antihypertensive medications (*n* = 252) attributed their behaviour in not taking the medication to being asymptomatic, and wrongfully thinking that absence of symptoms was a reliable indicator of a good blood pressure control. Other patients were noncompliant to treatment because they either experienced side effects (*n* = 8, 3.2%) or had financial restraints (*n* = 4, 1.6%), and some stopped taking medications because their BP was controlled (*n* = 20, 7.9%). The rest provided many other reasons for noncompliance including forgetting the medication, depression, and others.

Three hundred and thirty-seven patients (51.9%) were not complaint to salt restriction as they misunderstood it as only stopping pickles and spicy food.

Some patients (*n* = 219, 33.7%) claimed taking herbs as adjuvant to their antihypertensive medications, mostly hibiscus (*n* = 203, 92.7%) and to a much lesser extent doom palm and garlic (*n* = 39, 17.8%).

For the last question, 438 patients (67.5%) thought that follow up of HTN was important to avoid complications (*n* = 256, 58.4%), to ensure good BP control (*n* = 102, 23.3%) and to assess treatment efficacy (*n* = 76, 17.4%). After the phone call, most of these patients (*n* = 346/649, 53.3%) said that they intended to resume their office visits.

Patients who thought that follow up was not important (*n* = 180/618, 29.1%) explained their response by being asymptomatic (*n* = 45) and thus in no need for follow up.

## Discussion

Hypertension is a major public health problem as it affects about 30–45% of adults worldwide, and it is responsible for at least 9 million deaths every year [9]. The prevalence of hypertension is increasing over time because of aging of the population, tobacco smoking, sedentary lifestyle, and obesity. It is estimated that about 1.5 billion individuals will have hypertension by the year 2025 [10].

Hypertension is an important public health issue in Egypt. According to the Egyptian national hypertension project (NHP) in 1995, the estimated hypertension prevalence among Egyptians was 26.3%. Hypertension prevalence increased progressively with age, from 7.8% in 25–34-year-old to 56.6% in those 75 years or older. Only 37% of hypertensive population were aware of having high blood pressure, of those, only 23.9% were on anti-hypertension medications and only 8% had controlled systolic blood pressure < 140 mmHg and diastolic blood pressure < 90 mmHg [4, 5].

Hypertension represents a challenging public health problem because of its high prevalence, high risk of complications and its silent nature. Being an asymptomatic disease, HTN is probably diagnosed late as it was shown that patients tend to seek medical help only when they have symptoms. In addition, Egyptian patients lack the motivation for regular medical check-up and thus lots of hypertensive patients were discovered accidentally and late. After diagnosing HTN, absence of symptoms may give hypertensive patients a false impression of well-being and this may affect their compliance to clinic’s visits.

Egyptian specialized hypertension clinics (SHC) were established in 9 university hospitals and data of hypertensive patients presented to the SHCs in the period from January 2015 to December 2017 was recorded through online case report forms (CRFs).

This study included 3014 eligible patients whose data was provided by the online registry of the SHCs. Patients who attended more than 3 clinic visits within the registry period (12%) were considered compliant to follow up and were not telephoned. While patients who were not compliant to follow up visits (88%) were eligible to be approached through the phone and of those, only 24.5% could be successfully contacted and agreed to answer the questionnaire.

Non-compliant patients were generally younger, males, employed, had a higher educational level, and had a higher prevalence of cardiovascular risk factors and cardiovascular events as compared to patients who were compliant to clinics’ visits. Kotwani et al. [11] found that young men, and persons with low educational level were among those least likely to seek medical care.

It was observed that the presence of uncontrolled HTN was not a sufficient motive for follow up, as 91% of patients who had uncontrolled HTN in the first visit did not come for their scheduled next office visits. Regular follow up of hypertensive patients is important as a recent study showed that hypertensive patients who follow up regularly get their BP controlled more successfully and the regression analysis showed that regular treatment follow-up was an independent predictor of blood pressure control (OR 1.561 [95% CI 1.102–2.211; *p* = 0.024]) [8]. Another study from China showed similar results as they found a positive association between BP control and number of clinic visits [3].

Some patients explained non-compliance to clinics’ visits by their preference to measure their BP elsewhere, mostly in pharmacies. Patients preferred pharmacies as they are cheap, widely available, easily accessible at any time and with no long waiting periods. Yet, most patients reported wrong techniques during BP measurement in the pharmacy, where the BP was usually measured once, while they were standing and mostly without fully exposing the arm. A meta-analysis published in 2017 showed that the community pharmacy BP (CPBP) readings were generally higher than the clinic and the out-of-office BP measurements and the included studies exhibited important methodological differences in BP estimation. They concluded that the current evidence of CPBP measurement is inconclusive and should be interpreted with caution [12].

Apparently, the presence of symptoms was the main drive for compliance, as in this study, one of the most important causes of non-compliance to clinics’ visits was because patients were asymptomatic, as they thought that absence of symptoms meant a good BP control. Patients did not fully understand that hypertension is a silent disease, and they failed to perceive the risk of potential complications of HTN, which could include symptomatic and life-threatening conditions, such as coronary heart disease, chronic heart failure, stroke, or dementia. Cantillon et al. [13] and Kjellgren et al. [14] showed that about 50–92% of patients use their symptoms to estimate their BP levels. Other studies showed that wrong beliefs about symptoms associated with HTN could negatively affect the patients’ compliance to medications [15, 16]. From these studies we can conclude that a lack of knowledge about hypertension and its silent nature could be linked to suboptimal adherence to both treatment and follow up visits.

Another important reason of non-compliance to clinics’ visits was the long distance between the clinics and patients’ homes. Long distance and difficult transportation discouraged patients to attend the follow up visits and this was especially evident in patients coming from rural areas who needed several means of transportations. This was exhausting, and both time and money consuming. Thus, it is of utmost importance to provide a nationwide distribution of specialized HTN clinics so that patients would not have to travel for long periods to have their BP checked.

Financial status played a substantial role in patients’ compliance. As the SHCs did not provide neither free investigations nor free medications, patients were not eager to continue coming to HTN clinics. Added to this is the cost of transportation, and the absence of symptoms, patients preferred not to come to their scheduled office visits.

Fortunately, non-compliance to office visits did not affect compliance to medications, as 59.2% patients were compliant to their antihypertensive treatment despite non-compliance to office visits. This may be explained by the previously mentioned restrictions of cost, time, and distance of the clinics. Patients who reported non-compliance to medications gave many reasons, for example: being asymptomatic, experiencing side effects, forgetfulness, confusion due to taking many drugs, depression and inability to afford cost of treatment. Similar results were found by Youssef et al. [17] who used Logistic regression analysis to identify predictors of pharmacological compliance among hypertensive patients attending health insurance clinics (*n* = 316). They found that about 26% of the patients were non-compliant to medications and the common barriers to compliance were: feeling of controlled blood pressure, forgetfulness, and drug side-effects. In a study from Uganda, they found that some patients’ factors could affect the health seeking behaviour and these included: awareness of the disease, perceived severity of HTN, perceived effectiveness and adverse effects of medications, and fears of lifelong dependence on antihypertensive drugs [18].

Salt restriction is an important element in the dietary management of hypertension. The World Health Organization (WHO) showed that most people consume around twice the recommended maximum level of salt intake [19]. They also demonstrated that salt intake of less than 5 g per day for adults helps to reduce blood pressure and risk of cardiovascular disease, stroke, and coronary heart attack. Lower levels of daily dietary salt intake were recommended by The United Kingdom National Institute for Health and Care Excellence (3 g/day) and by the USA (4 g/day) [20].

In this study, most of the contacted patients were not compliant to salt restriction because they were not provided with the full list of dietary sources of high salt and they thought that cutting pickles off was enough for reducing their salt intake. This is fairly understandable because the time assigned to each patient in such a busy clinic was limited. Similar results were found by Youssef et al. [17] who showed that 22.4% of the patients attending the insurance hospitals were non-compliant to salt restriction.

About 33.7% of the contacted patients were taking complementary herbal treatment, mostly Hibiscus. Patients said that they were advised herbal treatment by one of the family members, neighbours, or friends. Use of herbal remedies was a common practice in hypertensive patients in Uganda, as well, but they mostly used garlic, ginger, pumpkin leaves, African eggplant (Katunkuma), aloe vera (kigaji), dried banana leaves, neem tree, chlorophyll extracts and human urine [18]. Similarly in Nigeria, patients considered herbal remedies as alternatives to antihypertensive medications [21].

Hypertension is a risk factor for both ischemic and haemorrhagic stroke [22]. In our study, 8% of the non-compliant patients had history of a previous stroke either ischemic or haemorrhagic. Some of those patients and other elderly patients were unable to come alone because of their disability and/or fragility. They needed family members to accompany them to the clinic, but sometimes, family members could not accompany them because of their work/familial obligations. In a recent review article, it was shown that poor quality of life and major disabilities were documented to negatively affect adherence to antihypertensive medications [23], especially when the medication(s) did not diminish the disability or improve the quality of life [2].

One of the reasons of non-compliance to follow up was that some patients preferred to follow up in private clinics because they thought that the level of care was better, and the timing of the clinic was more convenient. In South Africa, patients with higher economic resources were more likely to seek help from private doctors [24]. Other patients were originally referred to the cardiology and the SHCs to do some investigations as ambulatory blood pressure monitoring, echocardiography, coronary angiography, preoperative assessment, etc. and after getting the results, they continued to follow up with their private doctors. Patients who had insurance at other hospitals preferred to follow up their HTN in these hospitals instead of the SHC as insurance hospitals provided them with the required investigations and antihypertensive medications for free or at a very low cost.

Emotional factor could be a barrier to compliance to both follow up and treatment e.g. depression from loss of a loved one or frustration due to inability to achieve a blood pressure goal or to achieve a behavioural and a lifestyle modification caused some patients to stop coming to the clinics and to stop taking their anti-hypertensive treatment. That is why it is important to discuss with our patients, at each visit, the state of compliance and to ask about the reasons why they stopped their visits and/or their medications.

One of the most important reasons of non-compliance to follow up was the health care related factors as some patients were not satisfied by the provided healthcare service. Despite that most of the patients believed that their health care provider was competent and they trusted his/her skills, yet small number of patients showed some reservations due to either a bad experience with the healthcare provider or an unpleasant attitude of some of the clinic personnel. Some patients did not trust the healthcare provider as their treatment failed to control their symptoms. Many studies have shown a positive relation between trust of a physician and patients’ adherence to medications and lifestyle measures [25, 26]. In a study assessing health seeking behaviour of hypertensive patients in rural Uganda, it was found that the health systems factors influencing health seeking behaviour were related to availability and attitudes of staff and shortage of supplies and medicines [18].

Over-crowding and long waiting time were linked to non-compliance to follow up. This was attributed to the large number of patients in relation to the small number of health care providers. Many patients, especially the elderly found it exhausting to wait for long hours to get the service also it was hard for employed patients to take long leaves from their jobs to come to the clinics. Similar results were found by a study conducted in Barbados which evaluated the barriers of the primary care provided to hypertensive and diabetic patients (5 patients had HTN only, 5 had DM only and 11 patients had both diseases) [27]. They found that the main barriers of health seeking behaviour were difficulty of patients to maintain behaviour changes, difficulty taking time off work, failure of practitioners to consider the patient as a “whole person” and not showing enough respect for patients, long waiting times in public clinics and the high cost. They recommended primary healthcare providers to take a more patient centred approach to the care of patients with diabetes and hypertension and they recommended a better service by reducing the waiting times.

Kotwani et al. [11] conducted a community-based screening for hypertensive patients and they linked them to 5 focus care groups. They showed that the most common barriers for those who did not seek care included expensive transport (59%) and feeling well (59%). Similarly, Naanyu et al. [28] studied patients from rural areas of Kenya and evaluated the barriers of linking hypertensive patients to healthcare and had found 27 barriers, the most important of which were individual barriers (the asymptomatic nature of hypertension and the limited information about the disease) and environmental barriers, which included physical barriers (e.g. distance) and socio-economic barriers (e.g. poverty).

## Recommendations

- Physicians should keep a regular contact with their patients, as this attitude was shown to increase patients’ compliance to office visits and patients’ trust in the healthcare system.
- We need legislations to control the habit of measuring BP at pharmacies and we need to train pharmacists on the correct technique of BP measurement according to the latest guidelines.
- Patients need to be educated about HTN and its silent nature and they need to know that absence of symptoms does not mean that the BP is controlled.
- We need to provide our patients with a better understanding and demonstration of types of food that contain high salt content. So maybe we could provide fliers for educated patients and the attendant nurse may explain what is written in these fliers for patients who cannot read or write.
- While it is very difficult to change one’s beliefs, yet we need to explain to our patients that herbs are not yet approved for HTN treatment, and they should know that herbs are not a substitution to the antihypertensive medications.

## Conclusions

This study showed that adherence to the follow up visits in the Egyptian SHCs was poor due to many reasons. Absence of symptoms, overcrowding, long waiting times and long distance from home were the commonest reasons of non-adherence to clinic’s visits. Cost of transportation and cost of investigations and antihypertensive medications presented additional barriers against regular follow up. There should be community and healthcare campaigns to educate the patients about HTN and to explain the importance of adherence to medications and follow up visits.

## Data Availability

The datasets used and/or analysed during the current study are available from the corresponding author on reasonable request.

## Abbreviations

BMI: Body mass index
BP: Blood pressure
CAD: Coronary artery disease
CKD: Chronic renal disease
CPBP: Community pharmacy blood pressure
CRF: Case Report Form
DM: Diabetes mellitus
EHS: Egyptian Hypertension Society
ESRD: End-stage renal disease
HF: Heart failure
HTN: Hypertension
NHP: National hypertension project
OR: Odds ratio
SHC: Specialized hypertension clinics
WHO: World Health Organization

## References

[1] Antonakoudis G, Poulimenos L, Kifnidis K, Zouras C, Antonakoudis H (2007). Blood pressure control and cardiovascular risk reduction. Hippokratia.

[2] Burnier M, Egan BM (2019). Adherence in hypertension. Circ Res.

[3] Zuo HJ, Ma JX, Wang JW, Chen XR, Hou L (2019). The impact of routine follow-up with health care teams on blood pressure control among patients with hypertension. J Hum Hypertens.

[4] Ashour Z, Ibrahim MM, Appel LJ, Ibrahim AS, Whelton PK (1995). The Egyptian National Hypertension Project (NHP). Design and rationale. The NHP Investigative Team. Hypertension.

[5] Ibrahim MM, Rizk H, Appel LJ, el Aroussy W, Helmy S, Sharaf Y (1995). Hypertension prevalence, awareness, treatment, and control in Egypt. Results from the Egyptian National Hypertension Project (NHP). NHP Investigative Team. Hypertension.

[6] El Faramawy A, Youssef G, El Aroussy W, El Remisy D, El Deeb H, Abdel Aal A (2020). Registry of the Egyptian specialized hypertension clinics: patient risk profiles and geographical differences. J Human Hypertens.

[7] Abdel Aal A, Youssef G, El Faramawy A, El Remisy D, El Deeb H, El Aroussy W (2021). Registry of the Egyptian specialized hypertension clinics: sex-related differences in clinical characteristics and hypertension management among low socioeconomic hypertensive patients. J Clin Hypertens.

[8] Mahmood S, Jalal Z, Hadi MA, Shah KU (2020). Association between attendance at outpatient follow-up appointments and blood pressure control among patients with hypertension. BMC Cardiovasc Disord.

[9] W.H.O. Global Brief on Hypertension 2013. Available from: https://apps.who.int/iris/bitstream/handle/10665/79059/WHO_DCO_WHD_2013.2_eng.pdf;jsessionid=F638668E0D914847E4B72CCAFB90BC63?sequence=1.

[10] Kingue S, Ngoe CN, Menanga AP, Jingi AM, Noubiap JJ, Fesuh B (2015). Prevalence and risk factors of hypertension in urban areas of cameroon: a nationwide population-based cross-sectional study. J Clin Hypertens (Greenwich).

[11] Kotwani P, Balzer L, Kwarisiima D, Clark TD, Kabami J, Byonanebye D (2014). Evaluating linkage to care for hypertension after community-based screening in rural Uganda. Trop Med Int Health.

[12] Albasri A, O’Sullivan JW, Roberts NW, Prinjha S, McManus RJ, Sheppard JP (2017). A comparison of blood pressure in community pharmacies with ambulatory, home and general practitioner office readings: systematic review and meta-analysis. J Hypertens.

[13] Cantillon P, Morgan M, Dundas R, Simpson J, Bartholomew J, Shaw A (1997). Patients’ perceptions of changes in their blood pressure. J Hum Hypertens.

[14] Kjellgren KI, Ahlner J, Dahlof B, Gill H, Hedner T, Saljo R (1998). Perceived symptoms amongst hypertensive patients in routine clinical practice—a population-based study. J Intern Med.

[15] Alison Phillips L, Leventhal H, Leventhal EA (2013). Assessing theoretical predictors of long-term medication adherence: patients' treatment-related beliefs, experiential feedback and habit development. Psychol Health.

[16] Marshall IJ, Wolfe CD, McKevitt C (2012). Lay perspectives on hypertension and drug adherence: systematic review of qualitative research. BMJ.

[17] Youssef RM, Moubarak II (2002). Patterns and determinants of treatment compliance among hypertensive patients. East Mediterr Health J.

[18] Musinguzi G, Anthierens S, Nuwaha F, Van Geertruyden JP, Wanyenze RK, Bastiaens H (2018). Factors influencing compliance and health seeking behaviour for hypertension in Mukono and Buikwe in Uganda: a qualitative study. Int J Hypertens.

[19] World.Health.Organization. Salt reduction 2020. https://www.who.int/news-room/fact-sheets/detail/salt-reduction.

[20] He FJ, Tan M, Ma Y, MacGregor GA (2020). Salt reduction to prevent hypertension and cardiovascular disease: JACC state-of-the-art review. J Am Coll Cardiol.

[21] Singh N, Rahman SJ (2017). A study on the prevalence and awareness of hypertension among women in the reproductive age group and the factors contributing to it in a rural area of Jorhat district, Assam. Int J Community Med Public Health.

[22] Hägg S, Thorn LM, Forsblom CM, Gordin D, Saraheimo M, Tolonen N (2014). Different risk factor profiles for ischemic and hemorrhagic stroke in type 1 diabetes mellitus. Stroke.

[23] Holt EW, Muntner P, Joyce CJ, Webber L, Krousel-Wood MA (2010). Health-related quality of life and antihypertensive medication adherence among older adults. Age Ageing.

[24] Petrella RJ, Merikle EP, Jones J (2007). Prevalence, treatment, and control of hypertension in primary care: gaps, trends, and opportunities. J Clin Hypertens (Greenwich).

[25] Abel W, Efird J (2013). The association between trust in health care providers and medication adherence among black women with hypertension. Front Public Health.

[26] Jones DE, Carson KA, Bleich SN, Cooper LA (2012). Patient trust in physicians and adoption of lifestyle behaviors to control high blood pressure. Patient Educ Couns.

[27] Adams OP, Carter AO (2011). Knowledge, attitudes, practices, and barriers reported by patients receiving diabetes and hypertension primary health care in Barbados: a focus group study. BMC Fam Pract.

[28] Naanyu V, Vedanthan R, Kamano JH, Rotich JK, Lagat KK, Kiptoo P (2016). Barriers influencing linkage to hypertension care in Kenya: qualitative analysis from the LARK hypertension study. J Gen Intern Med.

